# Unpacking Online Discourse on Bioplastics: Insights from Reddit Sentiment Analysis

**DOI:** 10.3390/polym17060823

**Published:** 2025-03-20

**Authors:** Bernardo Cruz, Aimilia Vaitsi, Samuel Domingos, Catarina Possidónio, Sílvia Luís, Eliana Portugal, Ana Loureiro, Sibu Padmanabhan, Ana Rita Farias

**Affiliations:** 1 Digital Human-Environment Interaction Lab–HEI-Lab, Lusófona University, 1749-024 Lisbon, Portugal; emilyvaitsi@gmail.com (A.V.); samuel.domingos@ulusofona.pt (S.D.); catarina.possidonio@ulusofona.pt (C.P.); silvia.luis@ulusofona.pt (S.L.); eliana.portugal@ulusofona.pt (E.P.); ana.loureiro@ulusofona.pt (A.L.); ana.rita.farias@ulusofona.pt (A.R.F.); 2 School of Chemistry, Trinity College Dublin, D02 Dublin, Ireland; chullans@tcd.ie; 3 Advanced Materials and BioEngineering Research (AMBER) Centre, Trinity College Dublin, D02 Dublin, Ireland

**Keywords:** bioplastics, public response, sentiment analysis, social media analysis

## Abstract

Bioplastics have been presented as a sustainable alternative to products derived from fossil sources. In response, industries have developed innovative products using biopolymers across various sectors, such as food, packaging, biomedical, and construction. However, consumer acceptance remains crucial for their widespread adoption. This study aims to explore public sentiment toward bioplastics, focusing on emotions expressed on Reddit. A dataset of 5041 Reddit comments was collected using keywords associated with bioplastics and the extraction process was facilitated by Python-based libraries like pandas, NLTK, and NumPy. The sentiment analysis was conducted using the NRCLex, a broadly used lexicon. The overall findings suggest that trust, anticipation, and joy were the most dominant emotions in the time frame 2014–2024, indicating that the public emotional response towards bioplastics has been mostly positive. Negative emotions such as fear, sadness, and anger were less prevalent, although an intense response was noted in 2018. Findings also indicate a temporal co-occurrence between significant events related to bioplastics and changes in sentiment among Reddit users. Although the representativeness of the sample is limited, the results of this study support the need to develop real-time monitoring of the public’s emotional responses. Thus, it will be possible to design communication campaigns more aligned with public needs.

## 1. Introduction

The growing global concern about climate change and the depletion of fossil resources have led to worldwide efforts to find solutions that can mitigate their impacts [1]. Despite these efforts, global fossil-based plastic production has still been increasing, rising from 339.4 Mt in 2018 to 413.8 Mt in 2023 [2]. However, in Europe, fossil-based plastic production has slowly decreased from 62.3 Mt to 54.0 Mt over this same period, reflecting a trend towards reducing dependence on the shrinking reserves of fossil fuels [2]. In this context, the use of conventional plastics derived from fossil-based or petroleum-based feedstocks occupies a central barrier in the transition to a circular economy [3]. In fact, synthetic polymers have played a key role in society for over a century due to their physical and chemical properties, such as high durability, flexibility, and resistance to thermal variations [4,5,6]. These characteristics, combined with the low cost of production, have made these materials widely used across different sectors. Despite the benefits of these materials, the risks associated with their production and end-of-life management have raised many concerns, mainly due to carbon emissions and the consequences on the greenhouse effect, as well as their deposition in landfills causing pollution and microplastics’ poisoning of land and waterbodies [7,8,9]. It is well studied that the adoption of these materials may not only have an impact on the environment but also on the economy and health [10,11]. Bioplastics have emerged as a potentially sustainable alternative [12]. These alternatives to conventional plastics, according to European Bioplastics [13], can be conceptualized in three categories, which are bio-based (at least partially derived from biomass), biodegradable (capable of composting in appropriate facilities), or both [13,14,15]. Bio-based materials can be produced not only from sources such as plant biomass and agricultural waste (e.g., corn, sugar cane, or cellulose), but also from other non-fossil feedstocks (e.g., insects, mollusks, or food waste), being a major step towards achieving carbon neutrality and fossil resource independence [1,12,13]. Over recent years, they have increasingly been integrated into various market sectors, especially in packaging, agriculture, and medical applications, proving to be viable alternatives [16]. Although the general public is becoming increasingly aware of the need for this transition, there are still barriers on both the industry and consumer side that need to be explored regarding the adoption of these sustainable options [1]. With estimates that global plastic production could triple by 2060 [17], it is urgent to address the public’s unfamiliarity and ambivalent feelings towards bioplastics. A deeper understanding of these barriers could facilitate promoting greater levels of acceptance and conscious decision-making across different stakeholders. This paper reports on a Reddit-based sentiment analysis which we believe will contribute significantly to the body of work to promote bioplastics’ use in the coming years.

### 1.1. The Role of Consumer Emotions in Driving Industry Adoption of Bioplastics

Bioplastic production remains well below the desired volumes in comparison to conventional plastics. However, bioplastics have been gaining notoriety and market share, with a global forecast to grow from 1.813 million tons in 2022 to 7.432 in 2028 [13]. The production scalability of these sustainable materials seems to rely on three major categories of stakeholders: government; industry (production and commercialization); and consumers [9].

Despite the industry’s efforts to promote sustainable alternatives, government policies have been identified as barriers for a major shift towards bioplastics [18]. In this vein, consumer perception might be based on uncertainty, limited knowledge, and ambivalence towards these alternatives [19], and therefore consumers’ emotions might play a major role. Research has shown that when consumers are unaware of products they intend to buy, consumers’ emotions play a facilitating or inhibiting role throughout this process [20,21]. The affective processes (negative or positive emotions) associated with consumers’ personal and shared experience can thus have implications on their attitudes and beliefs, ultimately contributing to liking or disliking a product [22]. More specifically, positive affective responses associated with product characteristics such as price, quality, and safety seem to have an important influence on the intention to buy [23,24]. In this regard, a study [25] argued that consumers seem to seek not only a broader range of sustainable solutions but also more comprehensive product information. The use of complex or unfamiliar terminology can represent a barrier, leading to increased uncertainty and negative emotional responses [26,27]. This might be particularly evident in eco-labels and certifications, which, due to their complexity, can lead to misconceptions [18,28,29,30]. Previous studies [31] have found that while the term bio-based was recognized by many participants, few could explain what it meant and more than 70% were unaware of these materials’ benefits on waste reduction. Moreover, events such as greenwashing, where companies falsely claim environmental benefits, may be an important part of the explanation for the public’s uncertainty about sustainable solutions [32,33].

Understanding how these events may trigger the public’s emotional response could provide valuable insights to have a deeper approach in the generalized levels of uncertainty and mistrust towards green companies and green solutions. When analyzing consumer perceptions and attitudes towards new products and their associated beliefs, it is also crucial to understand how media portray these products and related events (e.g., climate movements and European conference agreements), knowing that it can shape public perceptions on the topic [34,35]. In this regard, the European Union has reported that Europeans’ main source of information on environmental issues is television news and social media. Scientific reports, on the other hand, seem to be one of the least used sources [36]. Given the growing use of social networks, debates promoted by the public and accessible online can be an important source to consider when analyzing how novel products, such as bioplastics, are being represented in online public responses.

### 1.2. Relevance of Online Platforms Like Reddit on Shaping Public Emotional Reactions to Bioplastics

Understanding consumers’ concerns, feelings, and expectations is crucial for promoting the adoption of bio-based materials. Public responses to sustainable products can, therefore, be an interesting bridge to explore underlying emotions. Considering the content of emotional responses in the public’s reaction enables a deeper analysis that goes beyond positive or negative emotions [37].

Many of the studies that have contributed to knowledge in this area have frequently used data collection methods through questionnaires, interviews, and focus groups [14,19]. These approaches, although validated and reliable, can have some limitations in the way they capture the public response. Among some of the limitations that may exist, the short representativeness in time and the change in participants’ behavior due to knowing that they are being observed (e.g., the Hawthorne effect) might be the most relevant [38,39]. In this regard, social networks have emerged as an important alternative to traditional data collection methodologies in contemporary research.

The exponential growth in user participation on digital platforms has sparked the interest of researchers in analyzing the content generated within these environments [40]. The increasing use of social media, with its large volume of qualitative and quantitative user-generated data, highlights the importance of incorporating this information into research to advance both theoretical and empirical understanding. Although the adoption of social network data as a research tool is relatively recent, its application across various domains has been growing [41]. The contribution of social media data to new insights is evident across multiple research fields, from social and political sciences to public health and behavioral studies [42,43].

Public and large-scale access to qualitative and quantitative data in real time has facilitated the use of content analysis techniques such as sentiment analysis. Sentiment analysis, a key method to assess public perceptions from user-generated content expressing beliefs and opinions, has become increasingly important due to its capacity to capture public opinion on political events and risk-related issues [44]. Social media data have the advantage of providing real-time, large-scale insights across diverse contexts [45]. This environment for data collection can be a valuable option for researchers in behavioral sciences and related fields, allowing deeper analysis of social dynamics, collective behavior, and public opinion [45]. Specifically, online platforms like Reddit have a unique anonymity mechanism, giving users enough safety to express their feelings and opinions [46].

Given Reddit’s accessibility and popularity [47], a growing volume of research has used it as a data source in the past decade [46]. Among some of its features, it allows users to make longer comments (e.g., compared to Twitter), has several levels of reply within the post, and allows upvoting/downvoting information. In addition to the inherent advantages of this platform, such as being topic-centered, it also has attractive characteristics from a research point of view, in which it provides longitudinal data that emerge in natural conversations. Due to the high volume of active discussions, Reddit uses moderators to manage misinformation and hate speech. While the platform uses strategies like quarantining (sensitive content alerts) and bans to avoid extremism and hate speech, there is always the risk of bias in moderators’ judgment about what comments cross acceptable lines [48,49]. Despite this control, perhaps in more sensitive topics (e.g., vaccination or political debate), the fact that users can freely choose to participate in any forum serves as a potential strength in the viability of using this data. Tapping into the thousands of comments made on this platform can thus provide important contributions to the development of new insights, enabling a better understanding of public responses while advancing scientific knowledge [46,50,51]. There seems to be a consensus among researchers about consumers’ feelings of uncertainty and lack of knowledge about bio-based and biodegradable materials, mainly due to the communication strategies used to date by governments and organizations [26,33]. However, there is a lack of research on how communication strategies regarding bioplastics impact public sentiment [52]. Moreover, online communities like Reddit have not been much used to study public sentiment towards bioplastics. Despite being widely used in quantitative approaches, few studies seem to have used qualitative or mixed-method approaches [30,35,53]. In this context, analyzing public emotional responses to bioplastics through Reddit presents a valuable opportunity. This approach allows a detailed analysis of how the public views bioplastics and how their responses have changed over the past few years. Public discourse on platforms like Reddit can provide unique insights, as it provides real-time consumer sentiments towards bioplastics and reveals how public events might be time-related to online public emotional variations. By analyzing such responses, this study seeks to identify which major emotions might underpin public responses towards bioplastics. This research intends to contribute to the broader understanding of how online discussions might provide public emotional response insights toward emerging sustainable technologies. Furthermore, this approach complements traditional survey-based research methods by addressing a gap in the literature related to the role of digital platforms in shaping consumer perceptions and the public debate surrounding sustainable materials.

## 2. Materials and Methods

### 2.1. Data Collection and Processing

Public sentiment towards bioplastics was assessed using user-generated comments on the topic extracted from the online platform Reddit. A total of 5041 comments were collected from 195 different posts, along with their titles, URLs, and comment dates. These posts were selected based on the presence of bioplastic-related keywords in their titles, ensuring their relevance to the topic. To extract the Reddit posts and comments, Python (Version: Python 3.13.2) was utilized in Google Collab, a free cloud service provided by Google that allows users to run Python code in real time using a browser-based environment (https://github.com/aimiliavaitsi/Biopliastics_Sentiment_Project/blob/main/Reddit_Web_Scraping_public.ipynb, accessed on 29 January 2025). Due to confidentiality reasons, sociodemographic data on sample characteristics could not be collected. Python was chosen due to its powerful libraries and common use in the research community for web scraping and sentiment analysis, previously validated by multiple studies [46,54,55,56,57,58,59].

To interact with Reddit’s API and extract data, we used PRAW (Python Reddit API Wrapper) due to its user-friendly design and integration capabilities. PRAW, when compared with other common APIs, such as Pushshift, simplifies query execution and response management, providing a more accessible and efficient solution for research workflows. PRAW requires developer enrollment in Reddit to obtain the client_id, client_secret, and user_agent credentials to access Reddit’s API. 

To ensure the quality of the Reddit comments, the PRAW (Python Reddit API Wrapper) library was used to fetch comments from posts containing specific keywords. The search queries included 32 keywords related to bioplastics, carefully selected to maximize the number of relevant comments. The final set of 32 relevant keywords can be seen in Table 1. The code ensured that the posts extracted from Reddit included at least one of the keywords below in their text to guarantee that the posts and comments were related to bioplastics. The top 5 keywords that contributed to our data extraction were bioplastic (38%), sustainable plastic (26%), biomaterial (19%), biodegradable plastic (7%), and bioplastics (4%). 

The script used a list (comments_data) to extract data, converting it into a pandas DataFrame. The DataFrame was saved to an Excel file using pandas and openpyxl. The final Excel file created by the script contained the following columns: post title, post text, comment, URL, and date (of the comment). To clean the data, Excel was used to eliminate duplicate comments. From 11,737 original comments extracted, 6.697 were duplicates. After excluding these, a total of 5041 unique comments remained. 

To validate that the post titles contained at least one of the 32 keywords in their text, a keyword frequency analysis was conducted in Excel. On average, each keyword appeared 2.07 times in the post titles, confirming that the script correctly filtered the titles. The dates of the posts and comments ranged from 2010 to 2024. The number of Reddit comments extracted per year is presented in Table 2.

Reddit’s community expanded rapidly from just over 10,926 subreddits in 2008 to 1.2 million in 2017 (source). Including earlier years in the analysis could make the data less reliable because there are too few comments (as also seen in Table 2 for the years 2010–2013), while using a shorter time frame might miss important trends in sentiment.

### 2.2. Sentiment Analysis

Sentiment analysis is defined as the study of sentiments, emotions, and opinions, as well as attitudes, towards a particular aspect in texts [60]. A typical sentiment analysis task involves determining whether a sentence expresses a positive or negative sentiment towards an aspect [61].

The present study aims to go beyond the traditional analysis of positive and negative polarity scores by examining emotions through Plutchik’s wheel of emotions framework [62]. In the 1980s, the author categorized emotions into eight groups, evenly split between positive and negative emotions. These emotions were joy, sadness, surprise, anticipation, trust, disgust, anger, and fear.

To achieve this, NRCLex was utilized in this study due to the user-friendly interface for applying the NRC Lexicon in Python. An emotion lexicon is a specific type of linguistic resource that maps the emotive or affective vocabulary of a language to a fixed set of emotion labels (e.g., Plutchik’s eight-emotion model), where each entry in the lexicon associates a word with zero or more emotion labels [63]. Sentiment lexicons may have different numbers of categories of emotion. NRCLex is an MIT-approved PyPI project by Mark M. Bailey [64]. It is used to predict the sentiments and emotions of an input text [64]. The dictionary contains around 27,000 words and is based on NLTK library’s WordNet synonym and the NRC Canada affect lexicon [64]. The lexicon is comprised of a list of English words, mapping eight elementary sentiments (joy, anger, surprise, fear, sadness, anticipation, trust, and disgust) and two polarities (positive and negative) [64]. The library assigns a list of emotions and their corresponding scores to the text based on the words associated with each emotion to perform emotion analysis [65]. Each emotion’s score, which runs from 0 to 1, indicates how strong that feeling was in the text [66]. NRCLex is a powerful tool used for the emotional analysis of huge amounts of text. It can be of great benefit in the fields of customer feedback, social media analysis, or market research. It has also been used broadly by the research community in various studies [65,66,67]. Finally, the lexicon is built through the crowdsourcing methodology, where each term was evaluated and annotated by five independent workers [68].

A new Google Collab script was employed for performing text preprocessing on the original Reddit comments, analyzing sentiment scores for each comment, normalizing the data, and visualizing emotional trends over a 10-year period of time (https://github.com/aimiliavaitsi/Biopliastics_Sentiment_Project/blob/main/Preprocessing_and_Sentiment_Analysis_per_Year_and_QTR.ipynb, accessed on 29 January 2025).

Using this script, initial data preprocessing was performed to tokenize the comments, converting them into lowercase, remove tokens that were not purely alphabetical (e.g., numbers and special characters), remove stopwords (e.g., and, the, is), and recombine the comments into a cleaned string for further analysis. NLTK (Version: 2024. NLTK 3.9.1) (Natural Language Toolkit) was used for preprocessing the data. NLTK (Natural Language ToolKit) is an open-source Natural Language Processing platform for Python [69], a tool that provides an easy-to-use interface with over 50 corpora and lexicon resources such as SentiWordNet (Version: WordNet 3.0) with a suit of text processing libraries for classification, tokenization, and semantic reasoning [69].

Due to the substantial variation in comment lengths, two sets of emotion scores were created: one with raw scores and another normalized by word count (see Supplementary Material for more information). As longer comments could disproportionately affect the emotion scores, this normalization helps balance differences in comment length. The normalization of emotion scores was performed using the following Formula (1), which provides a proportion or percentage that reflects the frequency of words associated with a specific emotion relative to the total number of words in a given comment:

(1) $$Normalized\;emotion\;score=\frac{\mathrm{Emotion}\;\mathrm{Word}\;\mathrm{Count}}{\mathrm{Total}\;\mathrm{Word}\;\mathrm{Count}}$$

The results, automatically computed by the script, were stored in a structured dataset. This dataset contained the following columns: Comment (the original Reddit comments), Year, Year-Quarter, Cleaned_Comment (the pre-processed text with unnecessary elements removed), Emotion_Scores (these columns contain numerical scores representing the number of words referring to these emotions for each comment), and Word_Count (showing the number of words in each preprocessed comment). Additionally, the file contained normalized emotion scores for each category: anger, anticipation, disgust, fear, joy, sadness, surprise, and trust, calculated using the formula previously stated.

The script generates various line charts based on both raw and normalized scores, as well as charts broken down by year and quarter. Due to the limited number of comments extracted for the years 2010–2013, as shown in Table 2, and due to not having comments for all the quarters from 2010–2013, these comments were excluded from the graphs to ensure a more reliable analysis. Including them could have skewed the results, as the small sample size might not accurately reflect sentiment trends for those years.

## 3. Results

After collecting and cleaning the extracted data, 5041 final comments from 195 different Reddit posts remained, using data from 2014 to 2024 due to the limited number of comments between 2013–2020 (see Table 2).

The average comment contains about 36 words. The most frequent comment length was just seven words, indicating a large number of very short comments. A median of 22 was found, indicating that most comments were shorter than 36 words. The standard deviation was 45.15, suggesting that there was a substantial variation in comment length. A sample of Reddit comments can be seen in Table 3.

**Figure 1:**
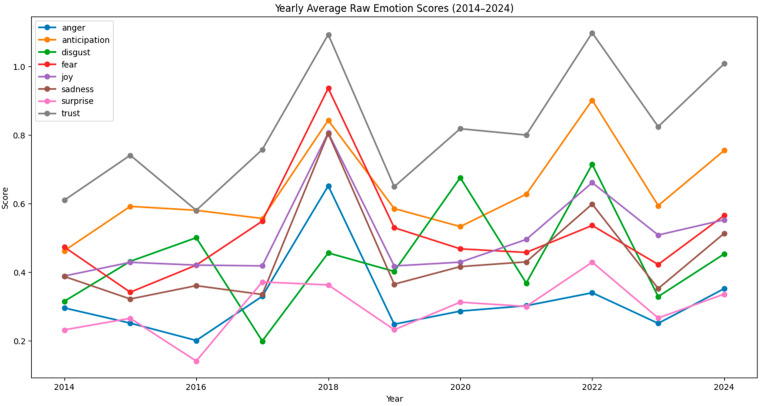
Emotion trends per year—raw data.

**Figure 2:**
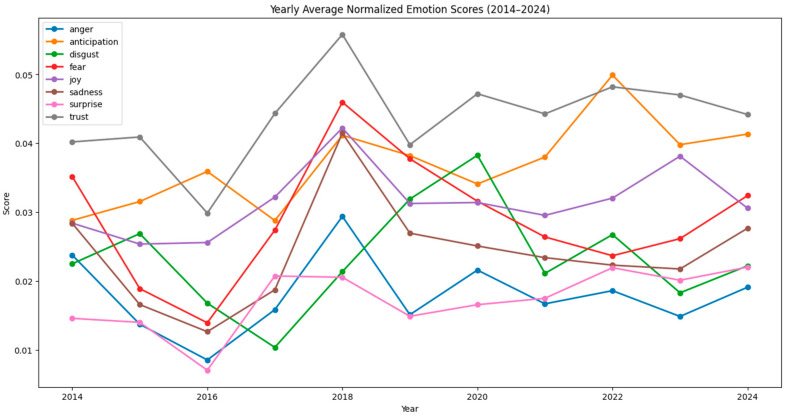
Emotion trends per year—normalized data.

**Figure 3:**
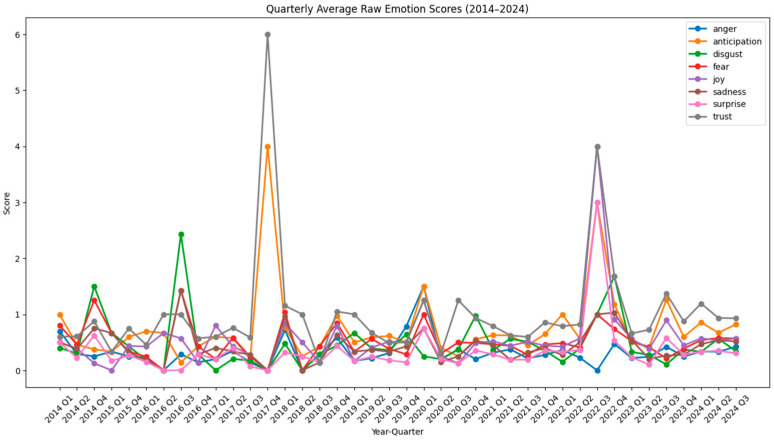
Emotion trends per quarter—raw data.

**Figure 4:**
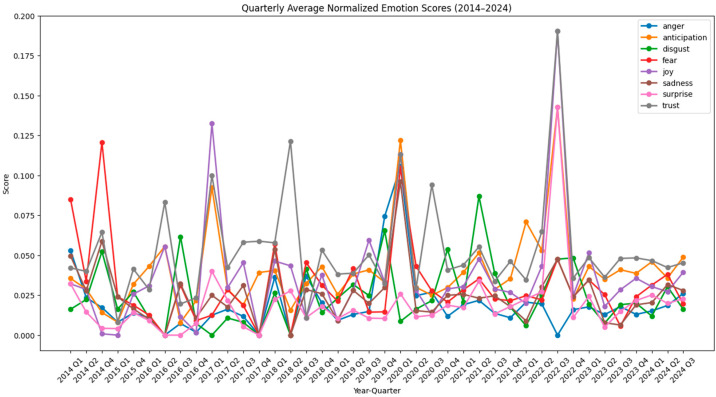
Emotion trends per quarter—normalized data.

Figure 1 and Figure 2 show raw and normalized results on the emotional trends in bioplastic-related discussions per year over a ten-year period, from 2014 to 2024. Figure 3 and Figure 4 show raw and normalized results on the emotional trends in bioplastic-related discussion per quarter over a ten-year period, from 2014 to 2024. The analysis was conducted using a sentiment analysis tool (NRCLex) that categorized emotions into eight categories: anger, anticipation, disgust, fear, joy, sadness, surprise, and trust. The sentiment scores were normalized to take into consideration the variations in word count, ensuring that the results were not being biased by changes in comment volume across the years. The resulting trends offer insights into public emotions around bioplastics during this decade.

Figure 1 and Figure 3, which display the raw yearly and quarterly emotion scores, have higher *y*-axis values because the scores are not scaled down. Unlike normalized graphs, these raw graphs do not account for differences in data size, which explains the broader range on the *y*-axis. Figure 1 shows sharper peaks compared to Figure 2 (for example, for the years of 2018 and 2022), since they are not adjusted for the total word count. In the yearly normalized graph (Figure 2), the *y*-axis represents the normalized emotion score, which is a proportion or percentage that reflects the frequency of an emotion relative to the total number of words associated with any emotion in that year. This scale ranges from approximately 0.01 to 0.06, indicating that emotions are expressed within a 1% to 6% range of all emotional expressions in any given year. The *x*-axis represents the years, ranging from 2014 to 2024. Even though comments may contain emotion-related words, they make up only a small fraction of the total number of words, which is why the normalized values are relatively low. Since emotions are measured separately, no single emotion is likely to dominate the entire text, which keeps individual proportions low. The peaks in Figure 2 are less extreme and the differences between emotions are more stable, since the word count is taken into consideration. Even though Figure 2 balances out these differences, the general trends remain similar to Figure 1.

In the quarterly normalized graph (Figure 4), the y-axis represents the normalized emotion score, which is a proportion or percentage that reflects the frequency of an emotion relative to the total number of words associated with any emotion in that quarter. Emotions scores range from 0.01 to 0.2 because scores are calculated in a shorter period (3 months), which leads to more extreme fluctuations.

The relative position of each line in the graphs reflects the overall prevalence of each emotion over time. Some emotions consistently show lower normalized scores, indicating they are less frequent or less dominant compared to others. Trust, anticipation, and joy (grey, orange, and purple lines) tend to be higher overall compared to other emotions, suggesting they are more frequently expressed across the data. Surprise (pink line) and anger (blue line) are often at the lower end of the chart, indicating that these emotions are expressed less frequently. Fear, disgust, and sadness (red, green, and brown lines) follow a similar pattern, fluctuating between moderate levels but rarely exceeding the peaks of emotions like trust or anticipation.

Overall, trust, anticipation, and joy are the dominant emotions, with their generally higher normalized scores indicating they have been expressed more frequently across time. All three of them were the most intense emotions in the years of 2016 and 2022 and decreased to a great extent by 2019. The year where negative emotions peaked was 2018. Fear, sadness, and anger experienced significant peaks during this year, indicating a period of negative sentiment and disapproval. In the most recent years (2023 and 2024), trust, anticipation, and joy were still the most dominant emotions. However, trust and joy slowly declined from 2023 to 2024, while all negative emotions (fear, sadness, anger, and disgust) slightly rose from 2023 to 2024. This trend may indicate growing skepticism or increasing concerns about the effectiveness, sustainability, or implementation of bioplastic solutions.

**Figure 5:**
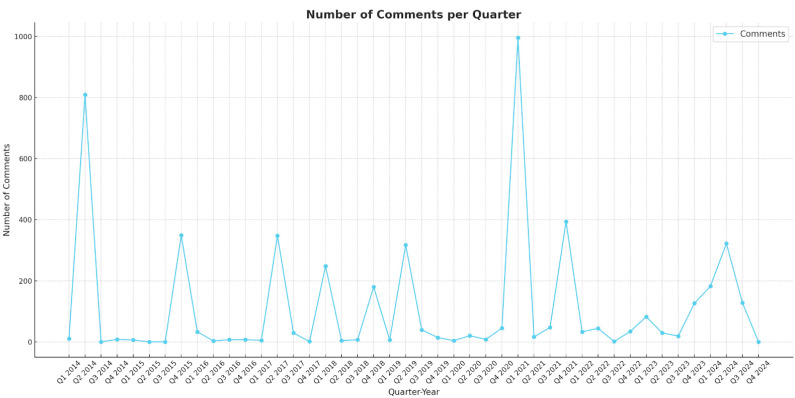
Number of comments per quarter.

The analysis of the number of comments extracted per quarter reveals interesting common patterns with significant environmental events (Figure 5).

For 2015, a total of 355 comments were extracted, with 349 of these occurring in Q4 of that year. This spike in comments aligned with the Paris Agreement [70], which was held in December of that year, suggesting that this important climate agreement may have increased public engagement on plastic-related topics. Additionally, in September of that year, slightly before Q4, the Sustainable Development Goals (SDGs) were adopted at the UN Summit [71], a very significant global milestone. The main emotions prevailing during Q4 of 2015 were trust, anticipation, and disgust, while the top words people were referring to were plastic(s), biodegradable, PLA, utensils, recyclable, and oil (see Supplementary Material). Similarly, for 2018, a total of 438 comments were extracted, with 248 comments recorded in Q1. This coincided with the introduction of the European Union Plastic Strategy in January 2018 [72], indicating that major policy announcements can significantly influence public conversation about environmental issues. The main emotions present during Q1 of 2018 were trust, fear, and sadness, while the main words people talked about were wound, healing, work, tissue, fetal, scar, cells, and blood. Moreover, of 376 total comments extracted in 2019, 317 of them were noted in Q2. This happened at the same time as the EU Single-Use Plastics Directive event in June of that year [73]. The main emotions prevailing during Q2 of 2019 were joy, trust, and anticipation and the main words people used were plastic(s), spider, glue, better, good, people, and cost. Finally, a last possible correlation is the one of 996 comments extracted in Q1 of 2021, coinciding with Biden’s administration rejoining the Paris Agreement in January of 2021 [74]. The most frequent emotions during Q1 of 2021 were disgust, trust, and anticipation and the most prevailing words were plastic(s), recycling, cost, oil, plant, and water.

Overall, the results indicate that people’s sentiment towards bioplastics was mostly positive for the time period of 2014–2024. The main sentiments that prevail in our dataset of 5041 Reddit comments are trust, anticipation, and joy. Negative emotions such as fear, sadness, and anger peaked in 2018. It is noticeable that quarters with high comments extractions coincide with major global environmental events (such as the Paris Agreement, the European Union Plastic Strategy, and the EU Single-Use Plastics Directive).

## 4. Discussion

In this study, a sentiment analysis was conducted on user-generated Reddit comments around the topic of bioplastics. Overall, the results are indicative that trust, anticipation, and joy are the dominant emotions, with their higher normalized scores indicating they have been expressed more frequently across time. All three of them were the most prevalent emotions in the years 2016 and 2022, decreasing to a great extent by 2019, with 2018 being the year where negative emotions were most prevalent. Fear, sadness, and anger showed significant peaks during 2018, indicating a period of negative sentiment and disapproval.

Moreover, the number of comments extracted by year reveals interesting theoretical correlations with significant environmental events. Interestingly, quarters with a higher number of comments co-occurred with major global environmental events, such as the Paris Agreement, the European Union Plastic Strategy, and the EU Single-Use Plastics Directive. These findings seem to suggest that it might be possible to analyze public emotional responses to economic and environmental events related to bioplastics through Reddit comments. Some events, such as the creation of European policy strategies, the 2030 Sustainable Development Agenda, and the Paris Agreement, seem to have driven positive responses, as the sentiment analysis results show. The intensification of these campaigns seems to align with the increase in comments about bioplastics, raising the question of whether there has been a greater awareness of bioplastic-derived product materials. However, the possible increase in public awareness levels, although relevant, does not necessarily imply an increase in the intention or behavior of buying sustainable products such as bioplastics. Some of the findings in this regard suggest an interesting peak in multiple emotional expressions at the same time frame, namely in 2019. Precisely during this peak, the Green Deal was established, thus reinforcing the hypothesis related to a possible relation between major events and public’s emotional response. When analyzing emotional fluctuations during this period, it is important to consider the social influence that climate protests had on the public, particularly in 2018 with the influence of public figures such as Greta Thunberg. Perhaps because of climate change movements and greenwashing scandals, anticipation has gradually become expressed more intensely in comments [75].

The media can also play an interesting role in the discussion, since the way it presents these events can encourage public debate about the topic and reinforce or change public psychological and behavioral responses [76]. Furthermore, based on the premise of the public’s uncertainty and lack of knowledge about these materials, how media portrays bioplastic-related events (e.g., as positive or negative) could have different implications for public emotional responses [77,78]. In other words, media coverage of major global environmental events can influence, amplify, and even change public psychological and behavioral responses. Precisely in this sense, there seems to be the need for more trustworthy information when it comes to sustainable products such as bioplastics (e.g., what makes it different from a conventional plastic product) and the need for more diversity in product offerings [25,31]. These strategies should promote consumer awareness and interaction, focusing on positive and meaningful experiences. This is because positive consumer emotional responses can be a powerful driver of demand, fostering the industry’s transition to the circular economy [79].

The findings of this study provide helpful lines of support for a better alignment between stakeholders and consumers within the bioplastics sector. Although the public’s emotional response is generally positive, the results seem to reinforce the need for clearer and trustworthy communication strategies that focus on consumers’ emotions. The stability and predictability shown when presenting new European directives appear to be sensitive to this need, as some of the most pronounced changes in emotional responses seem to be associated with these major events [80]. In line with what some studies suggest, close collaboration between companies and consumers can bring numerous advantages [81,82]. Sharing feedback between consumers and companies can not only boost positive emotional responses and trust in green solutions but also help in designing campaigns that are more focused on consumers’ needs and concerns.

Some of the findings reported in this study highlight the importance of monitoring public emotional responses in real time. The implementation of these models could bring numerous benefits, both in terms of supporting more effective communication strategies but also in promoting greater public acceptance of sustainable materials such as bioplastics. Nevertheless, there are some ethical concerns that should be considered, such as confidentiality of users, but also the need to ensure special care in any disclosure of data, given the significant risks involved.

Although this study provides relevant insights about the public’s emotional responses on Reddit, some limitations should still be considered. A major limitation of this study could be the representativeness of the sample since, for confidentiality reasons, Reddit does not disclose users’ personal data such as age or gender. Additionally, there is a potential risk that the dataset may not represent global sentiment due to potential biases in sampling. Some spikes might reflect algorithmic promotion or platform-specific activity rather than genuine public interest, potentially misinterpreting the results of public sentiment trends. Another limitation found in this study is the lack of a theory-based qualitative analysis, which could help to contextualize the results. It is unclear whether the negative emotions are associated with bioplastics or European policies. Similarly, regarding positive emotions, the distinction between bioplastic support or if they are for other events occurring at the same time it is unclear. Moreover, the lack of an ability to control the countries of the respondents could be another limitation. Hofstede’s analysis of cultural dimensions could add valuable insights into how emotions vary, considering some dimensions such as uncertainty avoidance and long-term orientation [83]. As another limitation, given the nature of online debates, public emotional responses may have been amplified by the course of the conversations [84].

Despite the findings presented in this study, several interesting questions have emerged that might be important to be explored in future studies. One such area would be to analyze whether the intensity of the public’s emotional responses differs between online and offline formats. Additionally, future research could explore experimental approaches in which emotional responses are manipulated to better understand how consumer reactions influence the adoption of bioplastic products. Moreover, examining public comments on bioplastics using advanced language models for real-time monitoring could lead to significant advancements in this research field. This approach could provide a deeper understanding of consumer responses, helping to optimize communication strategies and product development in alignment with public concerns and needs. These insights could be key in shaping more effective marketing strategies and policies based on consumer and ultimately contributing to a more widespread adoption of sustainable products. Addressing these questions could significantly contribute to a deeper understanding of public emotional responses and lead to a positive shift towards sustainable consumer behaviors.

## 5. Conclusions

This study provides a detailed analysis of the public’s emotional response towards bioplastics over 10 years. Through a sentiment analysis approach, important emotional responses were identified during this period. Overall findings suggest a positive public response towards bioplastics, reflecting some prevalent emotions such as anticipation and trust. However, peaks of negative emotional responses were also identified in 2018, with significantly higher levels of fear, sadness, and anger possibly associated with global environmental events. The findings also suggest that major climate events such as the Paris Agreement and the European Union Plastics Strategy had relevant effects on the volume of comments during the period.

This study underscores the importance of more effective communication strategies that address consumers’ emotions as a key point to build trust and increase bioplastic adoption. In this regard, close collaboration between companies and consumers could be a promising way to develop sustainable solutions more aligned with consumers’ needs. This study also identified some limitations, such as a lack of socio-demographic data, which may have limited the analysis to some extent, particularly concerning gender and age differences. Other possible limitations could be the amplification of public emotional responses in online debates. In this sense, it would be interesting to analyze whether there are indeed significant differences between public emotional responses in online and live formats.

## Figures and Tables

**Table 1:** List of relevant keywords used in search queries.

List of Keywords
biomaterial; biodegradation; biopolymers; bioplastic; biodegradable polymer; bioplastics; polyhydroxyalkanoates; biodegradable plastic; biodegradable materials; bio-composite; bio-based plastic; compostable plastic; bio-plastic; sustainable polymer; green plastic; sustainable plastic; PLA plastic; microbial plastic; renewable plastic; starch-based plastic; enzyme degradation; natural plastic; eco-friendly plastic; bio-derived plastic; marine biodegradable plastic; plant-based plastic; algae-based plastic; PHA plastic; polylactic acid; cellulose-based plastic; lignin-based plastic; chitosan plastic.

**Table 2:** Frequency and percentage of overall Reddit comments extracted per year.

Year	Count of Comments	%
2010	18	0.35%
2011	25	0.49%
2012	9	0.17%
2013	29	0.57%
2014	827	16.40%
2015	355	7.04%
2016	50	0.99%
2017	383	7.59%
2018	439	8.70%
2019	376	7.45%
2020	77	1.52%
2021	1453	28.82%
2022	112	2.22%
2023	256	5.07%
2024	632	12.53%
Total	5041	

**Table 3:** Sample comments from Reddit.

Post Title	Comment	URL	Date
“It’s greenwash”: most home compostable plastics don’t work, says study|Environment	The only solution to plastic waste is to stop relying on single use plastics. I think we’re too far dependent on the low cost and availability to go away from them now. All thats left is for humans to destroy the planet, annihilate themselves and let nature slowly come back over a few hundred thousand years. Then earth will be at peace once again	https://www.theguardian.com/environment/2022/nov/03/greenwash-home-compostable-plastics-dont-work-aoe	3 November 2022 17:59:38
Compostable plastic isn’t the green alternative you think it is	Banning the sale of plastic bottles would be a great first step to combat the plastic giants	http://sfchronicle.com/opinion/openforum/article/compostable-plastic-19570587.php	5 August 2024 20:42:44
97% of an algae-based plastic biodegrades in compost and water in under seven months	Self healing polymers might be a field of research what could eventually be combined with such bioplastics	https://www.reddit.com/r/conspiracy/comments/1bl5piq/97_of_an_algaebased_plastic_biodegrades_in/	22 March 2024 18:55:45
A bioplastic created from fish skin and scales and red algae could have a huge impact on limiting the amount of non-biodegradable plastic waste created in the world.	Until we run out of fish:/ Though at least the article says it’s repurposing a waste stream, so that is nice. Hopefully they stick to farmed fish if it catches on.	https://archive.sussex.ac.uk/news/press-releases/id/48861	23 August 2019 15:58:29
Sustainable, Eco-Friendly Plastic That Decomposes in Seawater	I believe it’s just better than the plastic we have now, which most don’t break down in the sea or provide food. So hopefully it continues to improve! Remember the ocean isn’t meant for garbage period, a lot goes there as most plastics aren’t recycled worldwide. Let’s see what more comes from this!	https://www.azom.com/news.aspx?newsID=62099	6 November 2023 14:25:14
Is Biodegradable Plastic Really a Thing? • Technically, it exists. But here’s what to think about when shopping.	Biodegradable options have existed for sometime now. The issue is the non-biodegradable ones are cheaper. Until economics change or companies are forced to do something nothing will change that.	https://www.nytimes.com/2024/05/20/climate/ask-nyt-climate-biodegradable-plastic.html	21 May 2024 09:14:32
Bioplastics as toxic as regular plastics; both need regulation, say researchers	Exactly, that’s what I agree with, but there’s a difference between bio-based plastics and biodegradable plastics. Something tells me biodegradable plastics aren’t nearly as toxic, possibly if at all. If they are, that would be good to know, but this feels like them lumping it all together to try to throw biodegradable plastics out with bio-based plastics. I smell big oil/big plastic in here somewhere.	https://news.mongabay.com/2024/04/bioplastics-as-toxic-as-regular-plastics-both-need-regulation-say-researchers/	24 April 2024 01:58:45

## Data Availability

The data presented in this study are openly available at: (https://doi.org/10.17605/OSF.IO/B49EY).

## Supplementary Materials

The following supporting information can be downloaded at: https://www.mdpi.com/article/10.3390/polym17060823/s1. Figure S1: Correlation between word count and emotions (heatmap); Table S1: Example of raw emotion scores; Table S2: Example of normalized emotion scores; Table S3: Descriptive statistics of the word count.
