# Supplementary material for: Unpacking Online Discourse on Bioplastics: Insights from Reddit Sentiment Analysis
Source: Polymers (Basel). 2025 Mar 20;17(6):823. doi: 10.3390/polym17060823 (PMC11945022; doi:10.3390/polym17060823)
Supplement: Supplementary file 1 [file polymers-17-00823-s001.zip › polymers-3479277-supplementary.pdf]

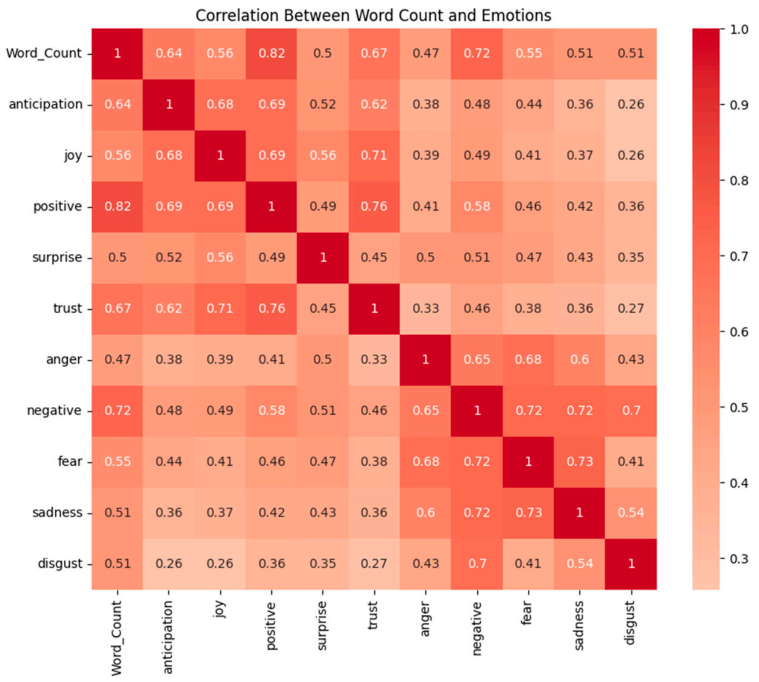

Figure S1. Correlation between word count and emotions (heatmap).

Table S1. Example of raw emotion scores.

Comment	Year	Word_Count	1	2	3	4	5	6	7	8
Example 1	2013	27	0	1	0	0	1	0	1	1
Example 2	2013	4	0	1	0	0	1	0	1	0
Example 3	2013	7	0	1	0	0	1	0	1	0
Example 4	2013	41	1	0	0	0	0	0	1	2
Example 5	2013	7	0	0	0	0	0	0	0	1

Note: **1**. Anger; **2**. anticipation; **3**. disgust; **4**. fear; **5**. joy; **6**. sadness; **7**. surprise; **8**. trust.

Table S2. Example of normalized emotion scores.

Comment	Year	Word_Count	1	2	3	4	5	6	7	8
Example 1	2013	27	0	0,0370	0	0	0,0370	0	0,0370	0,0370
Example 2	2013	4	0	0,2500	0	0	0,2500	0	0,2500	0
Example 3	2013	7	0	0,1428	0	0	0,1428	0	0,1428	0
Example 4	2013	41	0,0243	0	0	0	0	0	0,0243	0,0487
Example 5	2013	7	0	0	0	0	0	0	0	0,1428

Note: **1**. Anger; **2**. anticipation; **3**. disgust; **4**. fear; **5**. joy; **6**. sadness; **7**. surprise; **8**. trust.

Table S3. Descriptive statistics of the word count.

Statistic	Value
Mean	36.42134497
Standard error	0.643712524
Median	22
Mode	7
Standard deviation	45.70358923
Sample variance	2088.818068
Kurtosis	52.18755996
Skewness	4.997059673
Range	895
Minimum	1
Maximum	896

Additional Materials: Database of top 30 words per emotion

The following link directs to the script that identifies the top 30 words for each of the eight emotion categories: anger, anticipation, disgust, fear, joy, sadness, surprise, and trust. It also analyses the top 30 words for each of the eight categories per year and per quarter, for a deeper investigation in specific time frames.

https://github.com/aimiliavaitsi/Biopliastics_Sentiment_Project/blob/main/Top_30_Words_per_Emotion_category%2C_Year%2C_Quarter.ipynb
